# (*E*)-3-(1-Phenyl­ethyl­idene)indolin-2-one

**DOI:** 10.1107/S1600536811041316

**Published:** 2011-10-12

**Authors:** Qi Wang, Yue-Jun Zhang, Mao-Sen Yuan, Jun-Ru Wang

**Affiliations:** a College of Science, Northwest A&F University, Yangling 712100, Shannxi Province, People’s Republic of China

## Abstract

In the title mol­ecule, C_16_H_13_NO, the indoline-2-one ring system is nearly planar [maximum atomic deviation = 0.082 (2) Å] and is oriented at a dihedral angle of 66.60 (12)° with respect to the phenyl ring. In the crystal, inter­molecular N—H⋯O hydrogen bonds link the mol­ecules into supra­molecular dimers.

## Related literature

For applications of indoline-2-one and its derivatives as precursors in the synthesis of pharmaceuticals, see: Stephen *et al.* (1996 ▶).
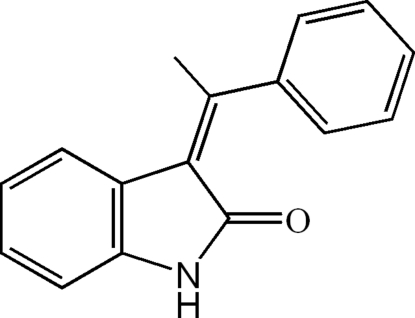


## Experimental

### 

#### Crystal data

C_16_H_13_NO
*M* *_r_* = 235.27
Monoclinic, 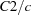

*a* = 22.215 (3) Å
*b* = 8.6259 (13) Å
*c* = 15.062 (2) Å
β = 122.097 (2)°
*V* = 2445.1 (6) Å^3^
*Z* = 8
Mo *K*α radiation
μ = 0.08 mm^−1^
*T* = 296 K
0.30 × 0.20 × 0.20 mm

#### Data collection

Bruker SMART 1000 CCD area-detector diffractometer
12693 measured reflections
2168 independent reflections
1599 reflections with *I* > 2σ(*I*)
*R*_int_ = 0.043

#### Refinement

*R*[*F* ^2^ > 2σ(*F* ^2^)] = 0.040
*wR*(*F* ^2^) = 0.125
*S* = 0.91
2168 reflections
165 parameters
H-atom parameters constrained
Δρ_max_ = 0.18 e Å^−3^
Δρ_min_ = −0.18 e Å^−3^

### 

Data collection: *SMART* (Bruker, 2001 ▶); cell refinement: *SAINT* (Bruker, 2001 ▶); data reduction: *SAINT*; program(s) used to solve structure: *SHELXTL* (Sheldrick, 2008 ▶); program(s) used to refine structure: *SHELXTL*; molecular graphics: *SHELXTL*; software used to prepare material for publication: *SHELXTL*.

## Supplementary Material

Crystal structure: contains datablock(s) global, I. DOI: 10.1107/S1600536811041316/xu5343sup1.cif

Structure factors: contains datablock(s) I. DOI: 10.1107/S1600536811041316/xu5343Isup2.hkl

Supplementary material file. DOI: 10.1107/S1600536811041316/xu5343Isup3.cml

Additional supplementary materials:  crystallographic information; 3D view; checkCIF report

## Figures and Tables

**Table 1:** Hydrogen-bond geometry (Å, °)

*D*—H⋯*A*	*D*—H	H⋯*A*	*D*⋯*A*	*D*—H⋯*A*
N1—H1⋯O1^i^	0.86	2.21	2.9002 (19)	137

Symmetry code: (i) 
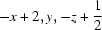
.

**Fig. 1.**
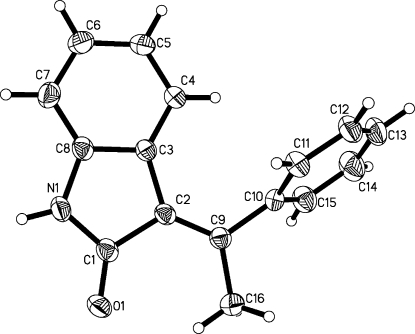
The molecular structure of the title compound with the atom numbering scheme. Displacement ellipsoids are drawn at the 30% probability level.

**Fig. 2.**
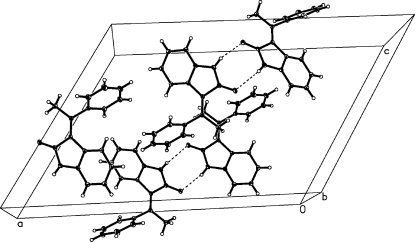
The molecular packing of (I) viewed along the c axis, with hydrogen bonds shown as dashed lines.

## supplementary crystallographic information

### Comment

Indoline-2-one and its derivatives have been used as precursors to synthesis pharmaceuticals (Stephen *et al.*, 1996). Rooting from its perfect conformation, indoline-2-one were tried to built electro-optic compounds recently. In the course of synthesis, we obtained the intermediate compound C_16_H_13_NO, (I), and the synthesis and structure are reported here.

In the title molecule, the indole system lies approximately in a plane and the maximum displacement from the least-square plane defined by all the 9 atoms of the indole framework is 0.082 (2) Å for C2 atom. The interplanar angle between the benzene plane and that of the indole moiety is 66.60 (12)°.

The title compound has three substituent ring systems, an indoline-2-one ring and two benzene rings which are arranged in a propeller-like fashion around the central atom C9 (Fig. 1). The interplanar dihedral angle between the two benzene rings defined by C10–C15 and C16–C21 is 73.41 (14)°. The interplanar angles between these benzene planes and that of the indoline moiety are 76.61 (12)° and 67.68 (12)°, respectively.

In the crystal structure there is an intermolecular N—H···O hydrogen-bonding interaction (Table 1) linking the molecules into dimers (Fig. 2).

### Experimental

Indolin-2-one (0.50 g, 3.76 mmol) was dissolved in THF (20 mL) and KOH (0.80 g, 14.3 mmol) was slowly added. After heating the stirred mixture at reflux temperature for 30 min, a solution of acetophenone (1.00 g, 8.33 mmol) in THF was slowly added and the refluxing continued for 2 h. The mixture was then cooled to 333 K and poured into water (200 mL) and was extracted with chloroform and dried over Na_2_SO_4_. After removing the solvent, the crude product was purified by column chromatography on silica gel, affording the title compound (yield: 0.15 g, 17%). The compound was then dissolved in THF, and yellow crystals were formed on slow evaporation at room temperature over one week.

### Refinement

All H atoms were placed in geometrically calculated positions with C—H = 0.93 (aromatic), 0.96 (methyl) and N—H = 0.86 Å, and refined using a riding model with U_iso_(H) = 1.5U_eq_(C) for methyl H atoms and 1.2U_eq_(C,N).

### Crystal data

**Table:**

C_16_H_13_NO	*F*(000) = 992
*M**_r_* = 235.27	*D*_x_ = 1.278 Mg m^−^^3^
Monoclinic, *C*2/*c*	Mo *K*α radiation, λ = 0.71073 Å
Hall symbol: -C 2yc	Cell parameters from 1702 reflections
*a* = 22.215 (3) Å	θ = 2.8–2.8°
*b* = 8.6259 (13) Å	µ = 0.08 mm^−^^1^
*c* = 15.062 (2) Å	*T* = 296 K
β = 122.097 (2)°	Block, colorless
*V* = 2445.1 (6) Å^3^	0.30 × 0.20 × 0.20 mm
*Z* = 8	

### Data collection

**Table:**

Bruker SMART 1000 CCD area-detector diffractometer	1599 reflections with *I* > 2σ(*I*)
Radiation source: fine-focus sealed tube	*R*_int_ = 0.043
graphite	θ_max_ = 25.1°, θ_min_ = 2.2°
φ and ω scans	*h* = −26→26
12693 measured reflections	*k* = −10→10
2168 independent reflections	*l* = −17→17

### Refinement

**Table:**

Refinement on *F*^2^	Secondary atom site location: difference Fourier map
Least-squares matrix: full	Hydrogen site location: inferred from neighbouring sites
*R*[*F*^2^ > 2σ(*F*^2^)] = 0.040	H-atom parameters constrained
*wR*(*F*^2^) = 0.125	*w* = 1/[σ^2^(*F*_o_^2^) + (0.0724*P*)^2^ + 1.3094*P*] where *P* = (*F*_o_^2^ + 2*F*_c_^2^)/3
*S* = 0.91	(Δ/σ)_max_ < 0.001
2168 reflections	Δρ_max_ = 0.18 e Å^−^^3^
165 parameters	Δρ_min_ = −0.18 e Å^−^^3^
0 restraints	Extinction correction: *SHELXTL* (Sheldrick, 2008), Fc^*^=kFc[1+0.001xFc^2^λ^3^/sin(2θ)]^-1/4^
Primary atom site location: structure-invariant direct methods	Extinction coefficient: 0.0027 (8)

### Special details

**Table:**

Geometry. All esds (except the esd in the dihedral angle between two l.s. planes) are estimated using the full covariance matrix. The cell esds are taken into account individually in the estimation of esds in distances, angles and torsion angles; correlations between esds in cell parameters are only used when they are defined by crystal symmetry. An approximate (isotropic) treatment of cell esds is used for estimating esds involving l.s. planes.
Refinement. Refinement of F^2^ against ALL reflections. The weighted R-factor wR and goodness of fit S are based on F^2^, conventional R-factors R are based on F, with F set to zero for negative F^2^. The threshold expression of F^2^ > 2sigma(F^2^) is used only for calculating R-factors(gt) etc. and is not relevant to the choice of reflections for refinement. R-factors based on F^2^ are statistically about twice as large as those based on F, and R- factors based on ALL data will be even larger.

### Fractional atomic coordinates and isotropic or equivalent isotropic displacement parameters (Å^2^)

**Table:**

	*x*	*y*	*z*	*U*_iso_*/*U*_eq_	
O1	1.03263 (7)	0.61802 (17)	0.39044 (11)	0.0561 (4)	
N1	0.91715 (8)	0.61084 (19)	0.25220 (12)	0.0472 (4)	
H1	0.9243	0.5627	0.2086	0.057*	
C2	0.93295 (9)	0.7148 (2)	0.40348 (14)	0.0390 (4)	
C3	0.85779 (9)	0.7304 (2)	0.31810 (14)	0.0403 (4)	
C8	0.85065 (9)	0.6645 (2)	0.22834 (14)	0.0426 (5)	
C10	0.92466 (9)	0.8083 (2)	0.55170 (13)	0.0414 (4)	
C1	0.96899 (10)	0.6443 (2)	0.35275 (15)	0.0429 (4)	
C4	0.79902 (9)	0.8025 (3)	0.30961 (15)	0.0515 (5)	
H4	0.8025	0.8502	0.3675	0.062*	
C9	0.96527 (9)	0.7516 (2)	0.50547 (14)	0.0411 (4)	
C11	0.87633 (10)	0.7128 (2)	0.55719 (16)	0.0510 (5)	
H11	0.8683	0.6128	0.5301	0.061*	
C7	0.78716 (10)	0.6622 (3)	0.13293 (15)	0.0546 (5)	
H7	0.7836	0.6156	0.0746	0.066*	
C16	1.04401 (10)	0.7398 (3)	0.58193 (16)	0.0580 (6)	
H16A	1.0643	0.6716	0.5543	0.087*	
H16B	1.0530	0.6995	0.6473	0.087*	
H16C	1.0651	0.8407	0.5931	0.087*	
C5	0.73531 (10)	0.8022 (3)	0.21390 (17)	0.0608 (6)	
H5	0.6959	0.8505	0.2079	0.073*	
C15	0.93593 (12)	0.9552 (3)	0.59383 (17)	0.0605 (6)	
H15	0.9691	1.0197	0.5929	0.073*	
C12	0.84032 (12)	0.7657 (3)	0.60250 (17)	0.0608 (6)	
H12	0.8085	0.7007	0.6066	0.073*	
C13	0.85102 (13)	0.9134 (3)	0.64162 (18)	0.0689 (7)	
H13	0.8260	0.9493	0.6710	0.083*	
C6	0.72902 (11)	0.7317 (3)	0.12704 (17)	0.0618 (6)	
H6	0.6853	0.7309	0.0640	0.074*	
C14	0.89866 (14)	1.0071 (3)	0.6371 (2)	0.0746 (7)	
H14	0.9060	1.1074	0.6636	0.089*	

### Atomic displacement parameters (Å^2^)

**Table:**

	*U*^11^	*U*^22^	*U*^33^	*U*^12^	*U*^13^	*U*^23^
O1	0.0469 (8)	0.0770 (10)	0.0553 (8)	0.0138 (7)	0.0345 (7)	0.0087 (7)
N1	0.0536 (10)	0.0554 (10)	0.0441 (9)	0.0045 (7)	0.0338 (8)	−0.0013 (7)
C2	0.0378 (9)	0.0435 (10)	0.0429 (10)	0.0001 (7)	0.0263 (8)	0.0033 (8)
C3	0.0385 (9)	0.0476 (11)	0.0402 (10)	−0.0018 (8)	0.0245 (8)	0.0020 (8)
C8	0.0464 (10)	0.0470 (11)	0.0430 (10)	−0.0040 (8)	0.0295 (9)	0.0011 (8)
C10	0.0411 (10)	0.0516 (11)	0.0322 (9)	0.0025 (8)	0.0199 (8)	0.0014 (8)
C1	0.0464 (11)	0.0473 (11)	0.0446 (11)	0.0029 (8)	0.0306 (9)	0.0068 (8)
C4	0.0421 (11)	0.0717 (14)	0.0455 (11)	0.0022 (9)	0.0265 (9)	−0.0023 (10)
C9	0.0404 (10)	0.0444 (10)	0.0420 (10)	−0.0006 (8)	0.0243 (8)	0.0037 (8)
C11	0.0524 (11)	0.0547 (12)	0.0561 (12)	−0.0002 (9)	0.0358 (10)	−0.0005 (9)
C7	0.0544 (12)	0.0707 (14)	0.0402 (11)	−0.0096 (10)	0.0261 (10)	−0.0037 (9)
C16	0.0412 (11)	0.0827 (15)	0.0466 (12)	0.0021 (10)	0.0210 (10)	0.0004 (10)
C5	0.0381 (11)	0.0907 (17)	0.0541 (13)	0.0038 (11)	0.0248 (10)	0.0030 (12)
C15	0.0648 (14)	0.0586 (13)	0.0652 (14)	−0.0076 (11)	0.0394 (12)	−0.0095 (11)
C12	0.0603 (13)	0.0780 (16)	0.0607 (14)	0.0080 (11)	0.0433 (12)	0.0105 (11)
C13	0.0740 (15)	0.0903 (18)	0.0589 (14)	0.0238 (14)	0.0464 (13)	0.0027 (13)
C6	0.0432 (11)	0.0912 (17)	0.0458 (12)	−0.0070 (11)	0.0201 (10)	0.0013 (11)
C14	0.0884 (18)	0.0674 (15)	0.0819 (18)	0.0027 (13)	0.0547 (16)	−0.0203 (13)

### Geometric parameters (Å, °)

**Table:**

O1—C1	1.233 (2)	C15—C14	1.373 (3)
N1—C1	1.360 (2)	C12—C13	1.370 (3)
N1—C8	1.401 (2)	C13—C14	1.362 (3)
C2—C9	1.343 (3)	N1—H1	0.8600
C2—C3	1.476 (2)	C4—H4	0.9300
C2—C1	1.498 (2)	C5—H5	0.9300
C3—C4	1.389 (2)	C6—H6	0.9300
C3—C8	1.396 (3)	C7—H7	0.9300
C8—C7	1.379 (3)	C11—H11	0.9300
C10—C15	1.379 (3)	C12—H12	0.9300
C10—C11	1.390 (3)	C13—H13	0.9300
C10—C9	1.485 (3)	C14—H14	0.9300
C4—C5	1.383 (3)	C15—H15	0.9300
C9—C16	1.502 (3)	C16—H16A	0.9600
C11—C12	1.376 (3)	C16—H16B	0.9600
C7—C6	1.383 (3)	C16—H16C	0.9600
C5—C6	1.381 (3)		
			
C1—N1—C8	111.66 (15)	C13—C14—C15	120.7 (2)
C9—C2—C3	130.16 (16)	C1—N1—H1	123.8
C9—C2—C1	124.99 (16)	C8—N1—H1	123.8
C3—C2—C1	104.85 (15)	C3—C4—H4	120.0
C4—C3—C8	118.39 (17)	C5—C4—H4	120.0
C4—C3—C2	133.92 (17)	C4—C5—H5	119.6
C8—C3—C2	107.50 (15)	C6—C5—H5	119.6
C7—C8—C3	122.84 (17)	C5—C6—H6	119.6
C7—C8—N1	128.17 (17)	C7—C6—H6	119.6
C3—C8—N1	108.93 (15)	C6—C7—H7	120.5
C15—C10—C11	118.31 (18)	C8—C7—H7	120.5
C15—C10—C9	120.74 (17)	C10—C11—H11	119.6
C11—C10—C9	120.91 (17)	C12—C11—H11	119.6
O1—C1—N1	123.98 (17)	C11—C12—H12	119.6
O1—C1—C2	129.26 (17)	C13—C12—H12	120.2
N1—C1—C2	106.76 (15)	C12—C13—H13	120.2
C5—C4—C3	119.10 (18)	C14—C13—H13	120.2
C2—C9—C10	121.69 (16)	C13—C14—H14	120.2
C2—C9—C16	123.92 (17)	C15—C14—H14	120.2
C10—C9—C16	114.40 (16)	C10—C15—H15	120.2
C12—C11—C10	120.2 (2)	C14—C15—H15	120.2
C8—C7—C6	117.63 (19)	C9—C16—H16A	109.4
C6—C5—C4	121.4 (2)	C9—C16—H16B	109.4
C14—C15—C10	120.7 (2)	C9—C16—H16C	109.4
C13—C12—C11	120.5 (2)	H16A—C16—H16B	109.0
C14—C13—C12	119.5 (2)	H16A—C16—H16C	109.0
C5—C6—C7	120.6 (2)	H16B—C16—H16C	109.0

### Hydrogen-bond geometry (Å, °)

**Table:**

*D*—H···*A*	*D*—H	H···*A*	*D*···*A*	*D*—H···*A*
N1—H1···O1^i^	0.86	2.21	2.9002 (19)	137

Symmetry codes: (i) −*x*+2, *y*, −*z*+1/2.
